# Lower air pollution during COVID-19 lock-down: improving models and methods estimating ozone impacts on crops

**DOI:** 10.1098/rsta.2020.0188

**Published:** 2020-09-28

**Authors:** Frank Dentener, Lisa Emberson, Stefano Galmarini, Giovanni Cappelli, Anisoara Irimescu, Denis Mihailescu, Rita Van Dingenen, Maurits van den Berg

**Affiliations:** 1 Directorate for Sustainable Resources, Transport and Climate, Joint Research Centre, European Commission, Ispra, Italy; 2 Directorate for Energy, Transport and Climate, Joint Research Centre, European Commission, Ispra, Italy; 3 Environment & Geography Department, University of York, Environment Building, Heslington, York, North Yorkshire YO10 5NG, UK; 4 Research Centre for Agriculture and Environment, Council for Agricultural Research and Economics, via di Corticella 133, 40128, Bologna, Italy; 5 Remote Sensing & GIS Laboratory, National Meteorological Administration, Sos. Bucuresti-Ploiesti, No. 97, Sect. 1, Bucharest 013686, Romania

**Keywords:** emission reduction, air pollution, ozone, COVID, crop production, wheat

## Abstract

We suggest that the unprecedented and unintended decrease of emissions of air pollutants during the COVID-19 lock-down in 2020 could lead to declining seasonal ozone concentrations and positive impacts on crop yields. An initial assessment of the potential effects of COVID-19 emission reductions was made using a set of six scenarios that variously assumed annual European and global emission reductions of 30% and 50% for the energy, industry, road transport and international shipping sectors, and 80% for the aviation sector. The greatest ozone reductions during the growing season reached up to 12  ppb over crop growing regions in Asia and up to 6 ppb in North America and Europe for the 50% global reduction scenario. In Europe, ozone responses are more sensitive to emission declines in other continents, international shipping and aviation than to emissions changes within Europe. We demonstrate that for wheat the overall magnitude of ozone precursor emission changes could lead to yield improvements between 2% and 8%. The expected magnitude of ozone precursor emission reductions during the Northern Hemisphere growing season in 2020 presents an opportunity to test and improve crop models and experimentally based exposure response relationships of ozone impacts on crops, under real-world conditions.

This article is part of a discussion meeting issue ‘Air quality, past present and future’.

## 1. Introduction

The unprecedented societal response to the ongoing COVID-19 pandemic has led to significantly reduced economic activities in the Northern Hemisphere since late winter and spring of 2020.

Lower levels of air pollution were reported throughout the period as a consequence of the shutdown of numerous activities and shifted or halted mobility and working patterns. Among the decreasing pollutants, NO_x_, the sum of nitrogen oxide (NO) and nitrogen dioxide (NO_2_), is the most important precursor of tropospheric ozone (O_3_), that in turn is toxic to crops, (semi-) natural vegetation and humans. At mid- and high-latitude regions of the Northern Hemisphere, O_3_ photochemical production is low in winter due to low sunlight conditions and temperatures, but increases rapidly in spring and summer. The lock-down has caused a reduction in the NO_2_ column by up to 30% in Europe and North America and by up to 50% in parts of Asia during spring 2020, as shown by satellite imagery (§2). Although O_3_ is not expected to decrease by the same proportion, such an abatement of NO_x_ will considerably reduce ground level O_3_ concentrations (§3) and O_3_ impacts on ecosystems, and potentially improve the productivity of crop, forests and grasslands.

Extensive evidence of O_3_ impacts on crops has been collected through controlled experiments during the past four decades [1]. These experiments have been used to develop exposure response relationships (ERRs). Application of these ERRs in risk assessment studies suggests that ambient levels of O_3_ across important agricultural regions cause yield losses to staple crops (wheat, rice, maize and soya bean). In Europe, this scientific evidence has supported the UNECE's Convention on Long Range Transboundary Air Pollution (CLRTAP) to establish critical levels for O_3_, which are essentially air quality targets for air pollution emission reduction policies. Despite the reduction of NO_x_ emissions by as much as 40% since 1990 in Europe and North America, these critical levels are still frequently exceeded. For instance, the recent CLRTAP assessment report [2] estimates current wheat yield losses due to O_3_ in Europe of the order of 13%.

A range of O_3_ and associated ERRs metrics exists to estimate crop losses [1]. In §3, we use the simple concentration-based AOT40 metric to demonstrate the potential benefits for crops of emission reductions during the COVID-19 lock-down. In §4, we explore the opportunity to gain additional insight into the validity of other concentration or flux-based metrics that have been developed to assess O_3_ damage [1], as well as more recently developed crop growth models that incorporate O_3_ effects.

The use of metrics to perform national and international O_3_ risk assessments stems mainly from the air quality impact research community and has not been mainstreamed into agronomic sciences. For instance, to our knowledge, no crop model used for operational crop yield assessments or crop forecasts incorporates the interaction between O_3_ and plant physiology. It remains to be determined whether the decrease in O_3_ exposure [2], as a consequence of the reductions of its precursor emissions in Europe, has led to increasing crop yields in recent decades. One of the main challenges is to isolate the overall benefits of O_3_ reduction on crop yields from other factors such as weather variability and management factors.

This paper suggests that the unprecedented and unintended COVID-19 lock-down in 2020 provides scientifically relevant information to quantify the actual O_3_ impact on crops. These unusual conditions caused an *in vivo* atmospheric experiment, whose magnitude could have generated sizeable reductions in surface O_3_ levels, and resulting increases in crop production in 2020. The subsequent analysis of agricultural statistics and application of O_3_ risk assessment and crop modelling will allow a comparison of the predictive ability of different methodologies to estimate regional-scale crop yield loss due to O_3_.

## 2. Are the observed nitrogen dioxide air pollution changes exceptional?

The best near-real-time information on emission changes is available for NO_x_. Emission changes of other O_3_ precursors are more difficult to derive from observations. The European Environment Agency (EEA) reports declining NO_2_ concentrations in several cities in Europe [3], as a consequence of the reduced activities associated with the COVID-19 outbreak. The data show consistent decreases in concentrations registered at road-side and background (sub-)urban monitoring stations from March to May 2020. The Copernicus Atmosphere Services (CAMS) also report reductions in NO_2_ concentrations [4], but caution that the use of highly variable time series of less than one month may lead to spurious conclusions on emission changes. Therefore, we focus on average values for March, April and May (MAM), with May the latest month available to us during the revision of this publication.

**Figure 1.**
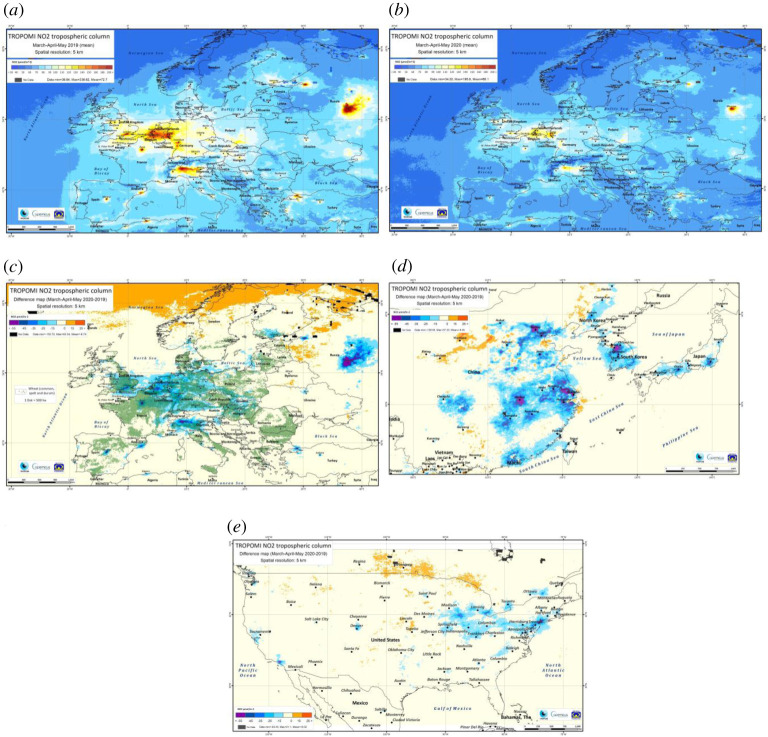
(*a*) TropOMI/Sentinel5P NO_2_ tropospheric column [µmol m^−2^] average for March–April–May 2019 over Europe (*b*) for 2020 over Europe (*c*) the difference of March–April–May 2020–2019 over Europe. The green areas indicate soft and durum wheat areas of 500 ha and larger (*d*) the difference of March–April–May 2020–2019 over Asia. (*e*) the difference of March–April–May over North America. (Online version in colour.)

Data from TropOMI/Sentinel5P (figure 1) show that persistent NO_2_ reductions in Europe were not confined to cities alone. We provide in the electronic supplementary material maps similar to figure 1, but for separate months to provide further insight in the temporal development of the NO_2_ reductions. Comparing MAM average NO_2_ columns in 2020 with 2019, large reductions are visible over extended regions of Europe, amounting to *ca*. 20% in Germany and the Benelux, 15% in Italy, 10–15% in Spain, France, the United Kingdom, Poland and Czech Republic, and 8% in Romania. Regions of emission reduction largely overlap with regions with extensive wheat production. Urban NO_2_ column reductions in Brussels, Dusseldorf and Paris are a few per cent higher than countrywide decreases, Milano's reduction of 27% is 10% higher than for Italy, and the 33% reductions in Madrid are markedly higher than the average 13% for Spain. There is significant uncertainty in estimating NO_x_ emission changes from 2020 to 2019 NO_2_ column changes, related to uncertainties in the satellite retrievals [5], the photochemical conditions of the atmosphere, but also due to inter-annual variability related to weather-related transport patterns. However, the changes in NO_2_ column in urban conglomerations and entire countries between 2020 and 2019 are clearly attributable to lock-down-related emissions variations, while smaller changes in cleaner areas can display residual inter-annual variability, which may obscure changes related to the COVID-19 lock-down.

In Asia, significant declines in MAM NO_2_ column by more than 50 µmol m^−2^ are found over several urban conglomerations (figure 1), corroborated by a similar analysis by ESA [6]. The largest declines in Asia are in March, with levels in April and May more similar to 2019 levels (electronic supplementary material S2). The MAM average emission reductions between 2020 and 2019 are of *ca*. 15% in Wuhan/Shanghai, 20% in Macao/Guangzhou, 20% in Tokyo, 18% in Beijing and Seoul—in March reductions of over 50% were seen.

In North America significant MAM averaged declines of 10–15% are also found over the Great Lakes, East and West Coast areas. In March, declines (up to 30%) were higher than in April and May (*ca*. 10%).

In Europe, these results can be compared to an earlier analysis of a step-wise emission decline of 20% in 2010 [7], which was at least in part due to a temporary reduction in emissions, resulting from the global economic recession in 2008–2009.

Therefore, we note that following this initial survey, which includes data up to the end of May 2020, further analysis over longer periods is needed to corroborate these column and related emission changes, and how these will affect ground level O_3_ concentrations. However, the compelling observational evidence of strong emission declines motivates our call to the wider research community to collect and analyse data on all related aspects of emissions, air quality and crop production.

## 3. What are the expected impacts on surface tropospheric ozone and crop production?

Surface O_3_ concentration depends on the magnitude and ratio of the emissions of precursor gases (e.g. NO_x_ and VOCs), photochemical reactions, atmospheric conditions (weather), removal processes at the Earth's surface and hence on local, regional and seasonal factors. In most regions, O_3_ declines with decreasing NO_x_ emissions; however, in some traffic-intensive urban regions dominated by high-NO_x_ emissions, the O_3_ response to declining NOx emissions may be initially positive, as reduced NO concentrations also reduce O_3_ titration close to sources, but as the plume of pollutants is transported away from urban areas net O_3_ production begins [8]. Detailed atmospheric chemistry transport models are generally used to evaluate the variety of regional responses to reductions in the mix of emissions. O_3_ can also be transported between regions within Europe and over longer intercontinental distances [9–13]. For instance, Jonson *et al.* [13] estimate that for a scenario assuming 20% reduction in global anthropenic emissions, up to 60% change in Phytotoxic Ozone Dose over forests (POD1) in Europe is due to changes in other regions. Therefore, it is likely that strong emission reductions in Asia and North America may also have influenced O_3_ in Europe.

To provide a gross estimate of the effects of the COVID-19 emission reductions on O_3_ air quality and its effects on crop production, we develop six illustrative scenarios. In scenario S1, NO_x_ and NMVOC emissions from transport, energy and industrial sectors are reduced by 30% in Europe. In scenario S2, the same reductions are applied worldwide. Scenarios S3 and S4 assume 50% lower emissions in Europe and the world, respectively. Scenario S4 is very similar to a recent O_3_-carbon cycle impact study [14], in which an emission reduction of 50% in these sectors was identified to dominate the overall positive impacts on Gross Primary Production of crops with a C3 metabolism (e.g. wheat). Scenarios S5 and S6 consider that emissions of international shipping were reduced by 30 and 50%, respectively, while international aviation emissions were down by 80% in both S5 and S6. As the exact timing of emission reductions is not known, for simplicity we assume in the scenarios year-round reductions, bearing in mind that for wheat the most O_3_-sensitive period is approximately during the grain-filling period (approximately end of May and June). We note that emission reductions of this magnitude or even higher are projected by 2050 if aggressive air pollution and climate polices are implemented [15,16], and changes during the COVID-19 lock-down may be indicative of the benefits of emission reductions projected over a much longer timescale. After the submission of this paper some more informal and peer-reviewed analyses on emission reductions have become available, which are now worth mentioning at the revision stage of this paper. The worldwide number of flights was sharply cut by 25% in March and by 60% in April and May 2020 [17]. Commercial shipping trade analysis [18] shows that in the five weeks after 12 of March 2020 the number of ship departures from major hubs declined by 6%, of which tanker traffic was mostly affected. Due to the slow response of the shipping sector to changing demand conditions these reductions are likely to become higher in the next months. A recent estimate [19] of impacts of the COVID-19 lock-down conditions on regional and global CO_2_ emissions showed that in April 2020 about 80% of the world's CO2 was emitted in regions affected by lock-down conditions, and the highest daily decline of global CO_2_ emissions was estimated for 7 April to range from −11% to −25% (central value −17%), with similar declines during the remainder of April 2020. Apart from residential emissions, all sectors analysed in [19] showed declines ranging from −7% for power generation to 60% for aviation, in agreement with the scenarios presented here. Depending on confinement levels (social distancing only, medium or stringent measures), surface transport emissions were reported down [19] by −10% to −50%, although numerous web reports also suggest larger reductions in selected cities. An overall annual impact on CO_2_ emission in 2020 was estimated [19] to range from −3% to −13%, with a central value of −7%, assuming a return to pre-COVID conditions in the middle of June 2020. Overall, the reported emission reductions support the choice of sensitivity studies presented in this publication, including the assumption that effects could very likely extend through June and July 2020. By a direct comparison with a variety of sources of information available at the time of the revision of this paper, we can conclude that scenarios S3, S4 and S6 are probably upper limits for the real-world impacts.

Some additional simplifying assumptions have been made in this study. For instance, we have not considered CO emission reductions, which could have some further minor impact on O_3_ formation [10]. Although the emissions of methane (CH_4_), another important O_3_ precursor, are probably also affected by the lock-down, and the reduction of air pollutants can influence the CH_4_ lifetime, the overall impact on CH_4_ and O_3_ concentrations is not a-priori clear. As any effect will play out on a timescale of 10 years (CH_4_'s lifetime), it will not likely be discernible within 2020, but may become substantial in the following years.

To estimate possible impacts of such emission reductions on crop yields, we use the TM5-FASST global source-receptor model [20]. In summary, TM5-FASST considers a set of 56 global regions and two global sectors (aviation and shipping), to determine the regional (grid-level) response to emission reduction of air pollutant precursor emissions. For each region and sector, the response of hourly O_3_ changes and corresponding impacts on crops is calculated using ERR available in the literature [20]. In this publication, we estimate impacts on crops using AOT40, which is a metric based on the cumulated concentration of hourly surface O_3_ above 40 ppb to which crops are exposed during a three-month period in the crop growing season. Comparison of TM5-FASST results with other studies described in [20] show coherent results among models and ERR methods, but the limitations of AOT40 and other ERRs should be noted, and are further discussed in [1].

**Figure 2.**
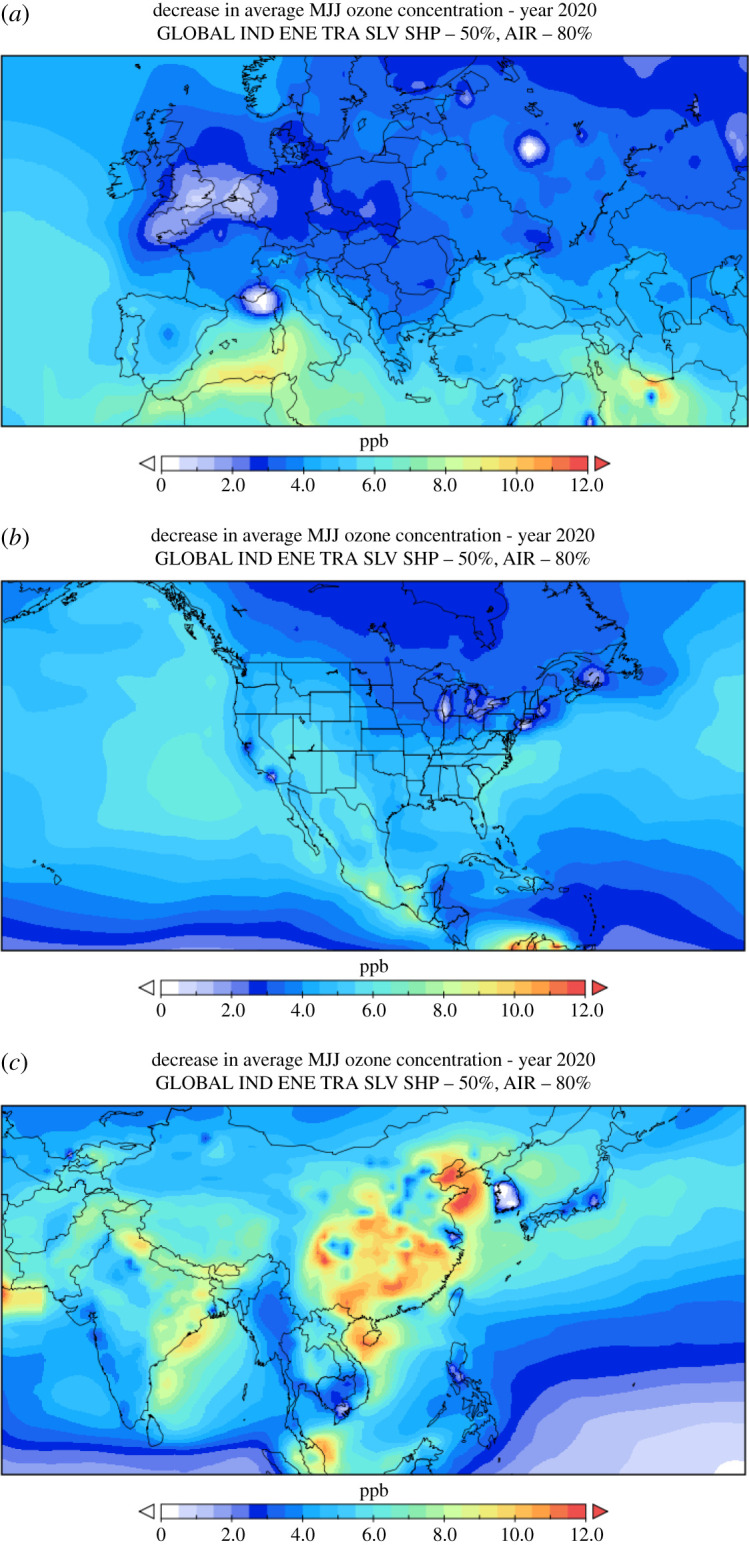
(*a*) Ozone responses (ppbv) calculated by TM5-FASST in Europe and Northern Africa, (*b*) North and middle America and (*c*) Asia (*c*) to global emission reductions by 50% in the industry, energy, transport, shipping and aviation (–80%) sectors, i.e. the sum of scenario S4 and S6. Isolated white regions correspond to near-zero or negative ozone responses due to declining emissions, which can occur in regions with high ratios of NOx to VOC emissions. (Online version in colour.)

Focussing on the scenario S4 + S6, the global 50% lower emission scenario, seasonal O_3_ changes in May–June–July (figure 2), range from 10 to 12 ppb O_3_ decreases in China and other parts of Asia, 2 to 6 ppb in North America, and less than 2 ppb in north-west Europe to up to 6 ppb in southern Europe. In particular, in northern Europe, and some other urban conglomerations in North America and Asia, emission reductions caused increases in O_3_. Accurate determination of O_3_ responses in such regions would need high-resolution models as well as high-resolution information on emission declines in those regions. For the 30% global emission reduction case (S2 + S5), O_3_ decreases were *ca*. 7 ppb in Asia, 1 to 4 ppb in North America and less than 1.5 ppb in Europe, respectively.

**Figure 3.**
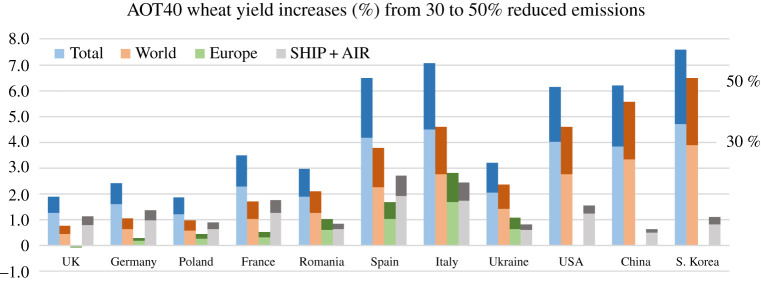
AOT40-based wheat yield increases (%) in selected European countries, USA, China and South Korea due to emission reductions by 30% and 50% in the energy, industry and transport sectors in Europe (S1, S3, green), world (S2, S4, blue) and international shipping + aircraft sectors (S5, S6 grey). The upper/lower part of the stacked bar represents the 50% and 30% emission reduction scenario, respectively, while aviation emissions were down by 80%. The total yield increase (blue) is the sum of the world and ship/aviation. Reference emissions were taken from the ECLIPSEv5a emission database [21] for the CLE-2020 scenario. Energy, industry and transport emissions amount to 56.6 and 6.2 Tg NO_2_ yr^−1^ for the world and Europe, respectively. International shipping and aviation emissions are 23.0 and 3.4 Tg NO_2_ yr^−1^, respectively. (Online version in colour.)

Impacts on wheat yields for the six scenarios are presented in figure 3. Overall improvements of wheat yields range from 1–4% in case of worldwide emission reductions of 30% (S2 + S5), and 2–7% for reductions of 50% (S4 + S6). The contribution of European emission reductions to yield improvements in a set of European countries is relatively small, ranging from approximately 0–0.5% in northern European countries to 3% in Italy (S1, S3). Shipping and aviation contribute up to 1–3% for scenario S6. Yield improvements of up to 7% were calculated for Asia and North America (S4 + S6).

## 4. There is an opportunity to learn about the real-world impacts of tropospheric ozone on crop production

Over the past few decades, a wide variety of concentration-based and flux-based O_3_ metrics have been used to develop ERRs for use in risk assessment studies. These studies have explored exceedance of critical levels, as well-estimated relative and absolute crop yield losses from anthropogenic O_3_ concentrations [1]. However, substantial differences in estimates of the magnitude as well as of the spatial distribution of yield losses have been found using different methods. For example, differences of up to a factor of two have been found when estimating yield losses using AOT40 versus POD metrics [22]. In addition, there are some long-term (5 to 10 years) statistical studies that try to identify the O_3_ signal in agricultural yield statistics by performing regression analysis of meteorological and O_3_ data. The results are not always consistent with empirically based risk assessment models [1]. This has led to uncertainty on the actual size of effect and spatial distribution of O_3_ on crop yields.

To address these uncertainties in risk assessment modelling, the Agricultural Model Intercomparison and Improvement Project (AgMIP) has started an activity to evaluate and enhance crop models with an O_3_ component. Some recent examples of the use of such crop models [23,24] show good potential to replicate O_3_ impacts found in field studies.

However, crop growth models used for operational agronomic analyses do not include O_3_ impacts. For example, the WOFOST model, currently used by the European Commission Joint Research Centre to provide operational analysis of crop growth development and yield forecasts [25], does not explicitly consider the effect of O_3_ on crop phenology and growth. In spite of this, the yield forecasts for which it is used can usually achieve an accuracy within ±3% [26]. This does not rule out the existence of an effect of O_3_ on yields, since such a signal is likely indirectly hidden in other climatic factors, e.g. air temperature and solar radiation [27], and can be removed during the post-calibration of the model results against a reference of historical yield data. If this is the case, we might expect that the explicit inclusion of O_3_ effects on crop development and yield in WOFOST and other crop models would further improve their performance for operational assessments, especially for those locations and years where O_3_ impacts can be high and vary between years. Understanding the combined impacts of future climate and air pollution projections further requires inclusion of O_3_ impacts in crop models.

COVID-19 has led to a myriad of societal consequences, including a strong decrease of economic activities, with grave impacts on livelihoods and society as a whole. Nonetheless, if the unintended in-vivo atmospheric experiments during COVID-19 result in substantial reductions in O_3_ and subsequent increases in crop productivity, it will allow us to evaluate and compare the different O_3_ metrics, risk assessment methods and crop growth models that have been developed. The new insights gained will support future development of operational agronomic analysis. Such analysis would need to be performed on careful consideration of other COVID-19 related factors (i.e. management decisions in response to expected returns) that may co-determine yields. Europe-wide, preliminary information [28] does not provide evidence of large-scale socio-economic responses by farmers, but this information needs to be corroborated at the end of the season.

Specifically, we see the following research opportunities and steps to take related to reduced emissions during the COVID-19 lock-down in 2020 and O_3_ impacts:

— The initial submission (5th of May 2020) of this publication intended to alert the research community to collect emerging data on emission and O_3_ changes and prepare atmospheric and crop models to perform an analysis of the role of O_3_ in determining 2020 crop yields. At the time of the revision of this publication (24th of June 2020), the latest observations indicated a continued emission reduction through the growing season, albeit with shifting regional importance as the SARS-COV-2 virus spreads around the world. Hence, extension of this analysis to other world regions would be advisable.
— Accurate estimates of emission changes in 2020 relative to the last 3–5 years, based on observed changes in NO_2_, statistical information from activities (e.g. fuel use changes, traffic information), and modelling multiple recent years. Better understanding of emission changes in specific sectors and reductions of other O_3_ precursors is important to understand overall impacts on emissions and O_3_. This paper showed the important role of intercontinental emissions, including shipping and aviation, which are therefore sectors that need particular attention. The approach to estimating CO2 emission reductions [19] may be extended and refined, taking advantage of the satellite observations of NO_2_ columns.
— Analysis and estimates of O_3_ changes due to lower emissions, focusing on the last 3–5 years, using current best available models, contrasted with available observations.
— Statistical analysis of agricultural yields from long-term experimental sites (to standardize management practices) over the past 3–5 years to assess whether emission reductions were sizeable enough to produce a significant yield anomaly in 2020.
— Evaluation of O_3_ risk assessment methods, using both concentration- and flux-based metrics, and crop models that incorporate an O_3_ component to assess their ability to predict changes in crop yields over the past 3–5 years.
— While the examples given in this publication focussed on wheat, evaluation of effects on other crops and ecosystems (e.g. grasslands and forests) known to be susceptible to O_3_ damage needs to be undertaken as well.

Following the methods developed in the UNECE CLRTAP Task Force on Hemispheric Transport of Air Pollution, and ICP Vegetation regarding hemispheric O_3_ modelling and impacts of ozone on vegetation and AgMIP modelling of ozone impacts on crops, coordinated modelling activities at the end of the 2020 cropping season can improve process understanding and model quality, ensuring the representation of the variety of modelling methods that currently exist.

## Supplementary Material

NO2 tropospheric column over Europe;NO2 tropospheric column over Asia;NO2 tropospheric column over North America

## Data accessibility

The TM5-FASST model is accessible at https://tm5-fasst.jrc.ec.europa.eu/. The Sentinel 5P L2 products are converted to L3 by the Google Earth Engine. Further data processing (monthly mean, monthly differences etc.) has been made using GEE APIs and code editor. Google Earth Engine was used in full compliance with the terms and conditions imposed by Google Inc. on usage rights.
