# Supplementary material for: Lower air pollution during COVID-19 lock-down: improving models and methods estimating ozone impacts on crops
Source: Philos Trans A Math Phys Eng Sci. 2020 Sep 28;378(2183):20200188. doi: 10.1098/rsta.2020.0188 (PMC7536037; doi:10.1098/rsta.2020.0188)
Supplement: NO2 tropospheric column over Europe;NO2 tropospheric column over Asia;NO2 tropospheric column over North America [file rsta20200188supp1.pdf]

PHILOSOPHICAL TRANSACTIONS A

Phil. Trans. R. Soc. A.
doi:10.1098/10.1098/rsta

Supplementary Material.
Lower air pollution during COVID-19 lock-down: improving models and methods estimating ozone impacts on crops.

Frank Dentener[1]*, Lisa Emberson[2], Stefano Galmarini[1], Giovanni Cappelli[3], Anisoara Irimescu[4], Denis Mihailescu[4], Rita Van Dingenen[5], Maurits van den Berg[1]

1 Directorate for Sustainable Resources, Joint Research Centre, European Commission, Ispra, Italy. ORCID ID: 0000-0001-7556-3076 (FD); 0000-0002-0321-152X (SG); 0000-0001-9584-4182 (MvdB)

2 Environment & Geography Dept., University of York, Environment Building, Heslington, York, North Yorkshire, YO10 5NG, UK. ORCID ID: 0000-0003-3463-0054

3 Research Centre for Agriculture and Environment, Council for Agricultural Research and Economics, via di Corticella 133, 40128, Bologna, Italy. ORCID ID: 0000-0003-4430-9838

4 Remote Sensing & GIS Laboratory, National Meteorological Administration, Sos. Bucuresti-Ploiesti, No. 97, Sect. 1, Bucharest 013686, Romania. ORCID ID: 0000-0002-0507-5277 (AI); 0000-0001-5831-2187 (DM)

5 Directorate for Energy, Transport and Climate, Joint Research Centre, European Commission, Ispra, Italy. ORCIDID: 0000-0003-2521-4972

Supplementary Material:

Figure S1 TropOMI/Sentinel5P NO_2 tropospheric column [μmol m^{-2}] over Europe monthly average for March, April and May 2019 and March, April and May 2020, and the difference of 2020 and 2019 for these months.

Figure S2 TropOMI/Sentinel5P NO_2 tropospheric column [μmol m^{-2}] over Asia monthly average for March, April and May 2019 and March, April and May 2020, and the difference of 2020 and 2019 for these months.

Figure S3 TropOMI/Sentinel5P NO_2 tropospheric column [μmol m^{-2}] over North America monthly average for March, April and May 2019 and March, April and May 2020, and the difference of 2020 and 2019 for these months.

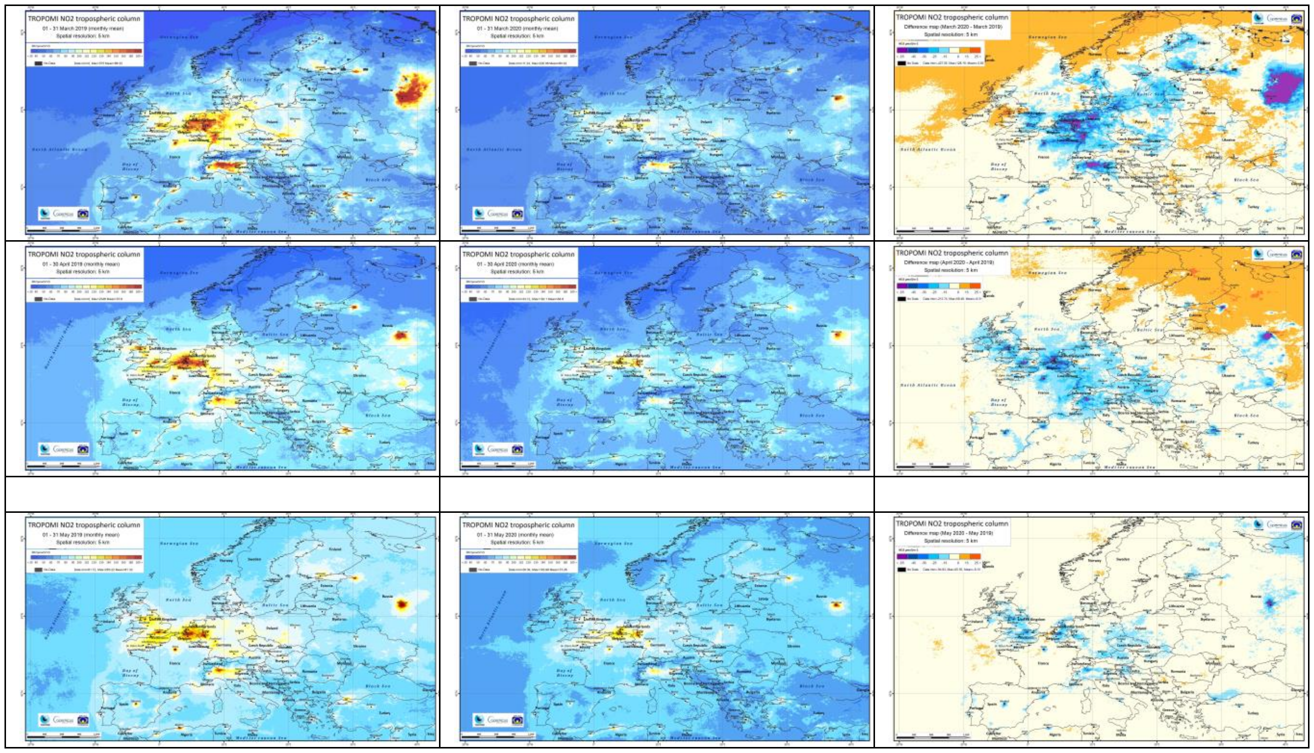

Figure S1: TropOMI/Sentinel5P NO_2 tropospheric column [μmol m^{-2}] over Europe monthly average for March (top), April (centre) and May (bottom) 2019 and March, April and May 2020, and the difference of 2020 and 2019 for these months.

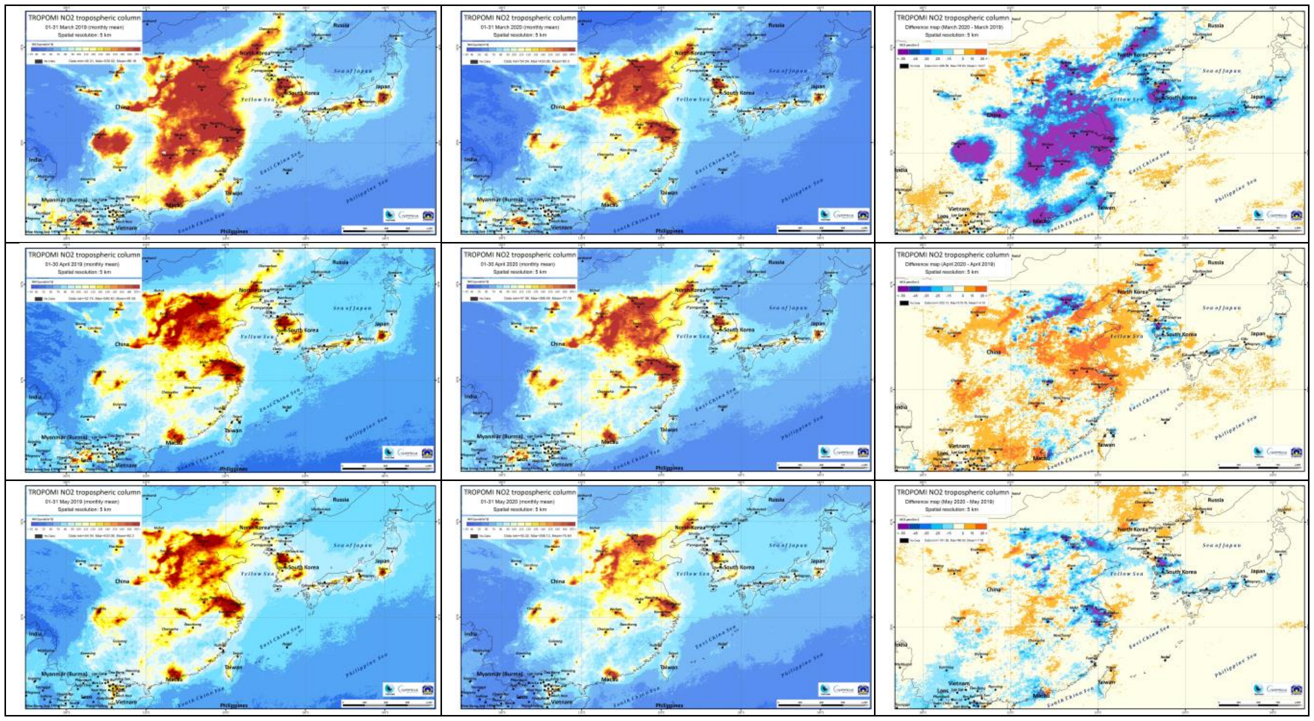

Figure S2 TropOMI/Sentinel5P NO_2 tropospheric column [µmol m^{-2}] over Asia monthly average for March (top), April (centre) and May (bottom) 2019 and March, April and May 2020, and the difference of 2019 and 2020 for these months.

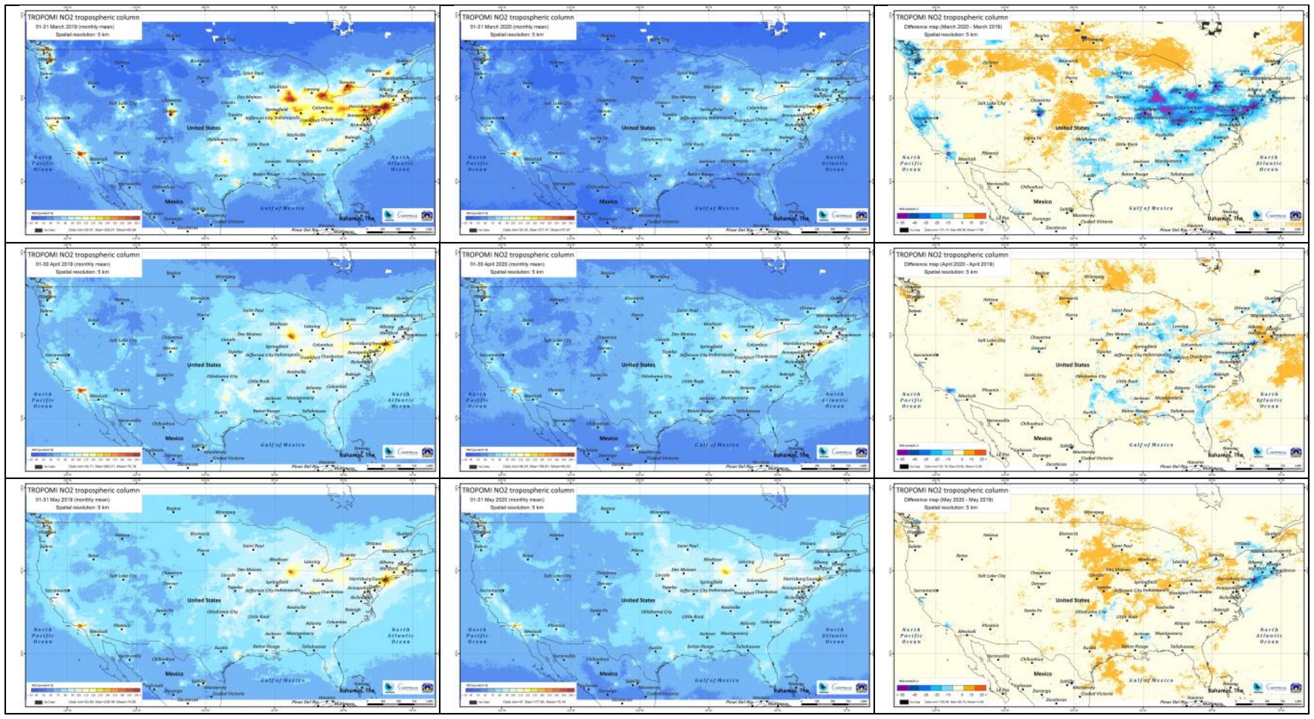

Figure S3 TropOMI/Sentinel5P NO_2 tropospheric column [µmol m^{-2}] over North America average for March (top), April (centre) and May (bottom) 2019 and March, April and May 2020, and the difference of 2019 and 2020 for these months.
